# Characteristics and purchasing behaviours of food-allergic consumers and those who buy food for them in Great Britain

**DOI:** 10.1186/2045-7022-3-31

**Published:** 2013-09-23

**Authors:** Stella Anne Cochrane, M Hazel Gowland, David Sheffield, René Wilfrid Robert Crevel

**Affiliations:** 1 Unilever SEAC, Colworth Science Park, Sharnbrook, Bedfordshire, MK44 1LQ, UK; 2 Anaphylaxis Campaign, Farnborough, Hampshire, GU14 6SX, UK

**Keywords:** Food allergy, Survey, Labels, Demographics, Diagnosis

## Abstract

**Background:**

Buying behaviours of food-allergic consumers can affect the risk they incur. An online survey was undertaken to understand the characteristics and buying behaviours of food-allergic consumers in Great Britain (GB) and people buying food for them.

**Methods:**

Descriptive study of food-allergic individuals in GB and their buying behaviours, based on a survey of 500 food-allergic consumers and 500 people buying for allergic individuals.

**Results:**

Fruit and vegetables were the most commonly mentioned food allergens for adults, cows’ milk in school-age children and eggs in younger children. 45% of respondents reported a formal diagnosis, almost half (48%) by a specialist. Significantly (P < 0.0001) more respondents reporting severe symptoms were likely to be formally diagnosed, but most reactions remained unreported. Nearly 2/3 of respondents always read product labels first time, however only 1/3 on every occasion. Only a third of respondents always avoided products with 'may contain’ labels. Respondents reporting severe symptoms, albeit still a minority, showed significantly (P = 0.0026) more cautious buying behaviours.

**Conclusions:**

Although self-reported, the pattern of food allergy reflects other studies. A minority of food-allergic individuals in GB, even among those reporting severe symptoms, have a formal diagnosis and most never come to the attention of health services, suggesting that food allergies are under-estimated while more severe reactors are over-represented in GB clinic populations. A substantial proportion of respondents regularly take risks when purchasing food including those reporting severe reactions, confirming that current application of precautionary labelling to mitigate and communicate risk is of limited effectiveness. Furthermore the failure of most food-allergic consumers to read labels on every occasion highlights the importance of thinking beyond legal compliance when designing labels, for example when adding an allergen to a product that previously did not contain it, the change should be flagged on the front of the pack to alert allergic consumers.

## Background

The prevalence of food allergy is reported to be rising in many countries, including Great Britain (GB), with approximately 3-4% of adults and 6-8% of children affected [1-5] and the prevalence of self-reported food allergy is even higher [6].

Although immunotherapy for food allergies is progressing [7-9], there are still no widely available long-term curative treatments available for people with food allergies, and as such they must manage their condition through careful avoidance of their trigger foods and use of medications to treat any arising symptoms when avoidance fails.

European Union legislation mandates the declaration of 14 major allergens (milk, egg, fish, crustacea, molluscs, lupin, peanuts, tree nuts, cereals containing gluten, sesame, soya, celery, mustard and sulphites) and their derivatives (unless specifically exempted) at present when used as ingredients in pre-packed foods and in clear terminology [10-12]. However guidance regarding unintended presence of allergens, for instance through cross-contact is less clear and has led to widely varying practices and terminology. As a result, confusion and issues of trust and interpretation have become prominent in relation to precautionary labels such as 'may contain’ or 'manufactured on a facility that handles tree nuts’ [13-18]. As a result much effort has been and continues to be invested in defining a risk-based approach to the assessment and management of unintentionally present allergens in foods, including their labelling [13,14,19-21].

This situation is aggravated by the lack of specialist advice, which is often difficult for patients to access in many countries, including GB [22-24]. Individuals therefore rely on self-diagnosis in interpreting their symptoms and attributing them to particular culprit foods, and self-education regarding food avoidance. This can result in serious consequences relative to food choices, nutrition and the risk to which they are exposed.

The aim of this study was to ascertain the characteristics and experiences of members of the general public who consider themselves to have food allergy and/or who buy food for allergic individuals.

As the buying behaviour of food-allergic consumers can affect the risk that they incur, the results of this survey can be used to improve safety for these consumers by gaining insights into attitudes to risk and how it is managed.

## Methods

An online survey was designed with a filter question to focus upon those people experiencing symptoms typical of 'true’ food allergy, and delivered with the aim of surveying 500 food allergic individuals and 500 people buying for such individuals.

The filter question used was: *'Symptoms of allergic reactions to foods can include mouth itching, swelling of the lips, face, throat, mouth and/or tongue, rashes, asthma, or even collapse and unconsciousness. These reactions almost always happen within two hours of eating the food’. Have you ever suffered any such reaction to any foods, or are you the main food buyer for someone who has suffered from such a reaction? Please tell us even if the reaction was relatively mild.*

The full question set covered in this publication is provided in an additional file [see Additional file 1].

The survey was not aimed at any selected population, such as members of allergic patient groups, but delivered with the aim of reaching a group of people as representative of the wider, general population in Great Britain as possible. An online research agency was therefore approached to deliver the questionnaire to an unselected panel of respondents who had opted in to participating in online research activities. No specific requests were made to target specific groups such as members of an allergy support group. Additionally no guidance was provided to respondents regarding any of the questions or terminology used and thus the responses given reflect the personal understanding by the respondents of terms such as anaphylactic shock and so forth.

As the survey was delivered by a third party, an online market research agency, none of the authors had access to the primary/personally identifiable data.

Results were analysed with SAS v.9.3 using the procedure PROC FREQ. This procedure produces multi-dimensional frequency tables for count data. Where comparisons were undertaken a Chi Squared Test for association was used.

## Results and discussion

A total of 949 respondents (537 food allergic individuals and 501 food buyers (including 89 of the food allergic individuals)) completed the online survey. 55.32% of respondents were female, 44.47% male and the population surveyed was close to being nationally representative with respect to age distribution (18% aged 18–29 years, 51% aged 30–50 years and 31% aged over 50 years), regions (23% from London, 24% the rest of southern England, 21% Midlands and Wales, 22% the North of England and 8% Scotland) and socio-economic groups (67% in income bracket ABC1 (middle class) and 33% in C2DE (working class)).

### Food allergy demographics

When asked 'Who in your household suffers from food allergies?’ the highest incidences of food allergies were reported in children (65%), with 34% affecting school-age children (5–17 years) and 31% affecting younger children (0–4 years). The remaining 35% of allergies were reported to affect adults in the household.

**Figure 1:**
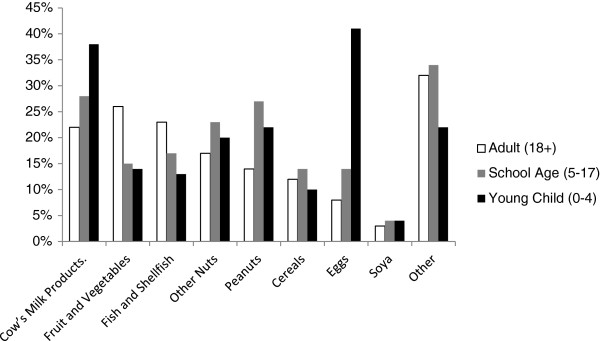
Foods reported as responsible for allergies, grouped by the age of the individual affected (Percentage of all respondents (n = 949)).

For all respondents (n = 949), as shown in Figure 1, fruit & vegetables were reported as the main cause of food allergy in adults (26%), while cows’ milk was most common in school age children (28%) and eggs in younger children (38%). Nearly a third of respondents (31%, n = 295) selected the 'other’ category of food groups as responsible for causing allergy [Additional file 1], with 41% (n = 121) of these respondents selecting this category in addition to one of the other eight. A wide range of food stuffs was described with the largest group being specified fruits and vegetables, which should have been captured under option 8 (fruit and vegetables) but were not, perhaps reflecting a lack of understanding of where some foods are categorised or a wish to be more specific. In the fruit and vegetable group the largest number of responses described alliums such as onions and garlic, followed by citrus fruits, chillies/peppers, bananas and then tomatoes. After fruit and vegetables the next largest food group described was additives (colorants, preservatives, flavourings, monosodium glutamate and artificial sweeteners etc.) followed by meats, further foods which should have been captured under options 1–7 (11 referring to wheat, gluten or bread, 5 to specific fish or shellfish and 4 referring to tree nuts), then chocolate, wine, spices, sesame seeds and legumes other than peanuts. A relatively large number of respondents (n = 23) also indicated that their trigger food had yet to be identified and in some cases it was also conveyed that this was a cause of distress.

**Figure 2:**
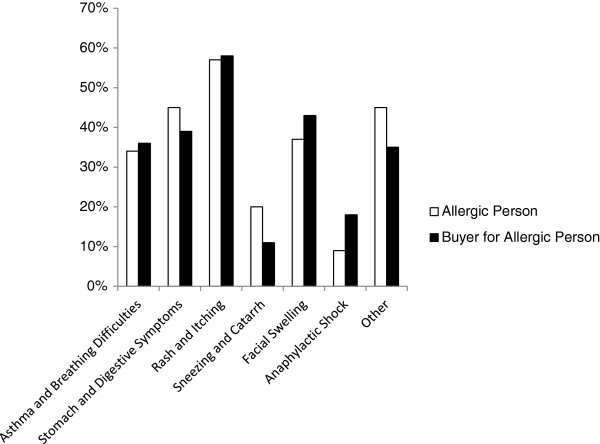
Symptoms of food allergy reported by the two main groups of respondents (respondents separated into two mutually exclusive groups: food allergic individuals (n = 537) and food buyers only (n = 412)).

Overall the most frequently reported symptoms were rash and itching, followed by stomach and digestive symptoms, then asthma and breathing difficulties (Figure 2). For cow’s milk, eggs, fish and shellfish and fruit and vegetables, rash and itching were the dominant symptoms. Reactions to peanuts and other nuts were associated with rash and facial swelling while stomach cramps and other digestive symptoms were predominant with soy and wheat. When asked 'What type of reaction(s) have you/ your family member suffered? [Please tick all that apply]’ , of the 16 options available, anaphylactic shock was selected by a relatively high proportion of all respondents (13%) and more so by those buying for an allergic person than allergic people themselves (9% (50/537) versus 18% (75/412) respectively) (Figure 2) (For this analysis respondents were separated into two mutually exclusive groups: food allergic individuals (n = 537) and food buyers only (n = 412)). Anaphylactic shock was most commonly selected by respondents citing allergy to peanuts (28% (44/155)), other nuts (30% (53/174)) and soy (25% (7/28)).

### Diagnostic demographics

**Figure 3:**
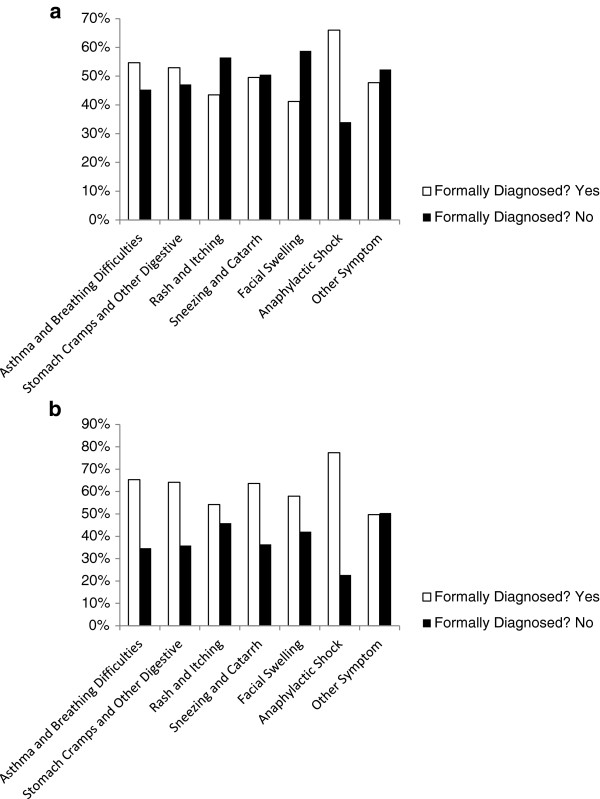
Comparison of reported food allergic symptoms described by respondents reported to be formally diagnosed and not formally diagnosed with food allergy and presented separately for food allergic individuals (a) and those buying foods for such individuals (b).

45% of all respondents reported their allergy as being formally diagnosed, with 54% of these indicating diagnosis by a general practitioner and 48% by a National Health Service (NHS) specialist. Respondents reporting severe symptoms were more likely to be formally diagnosed than those with mild symptoms, whether this reporting was by food allergic individuals (Figure 3a) or those buying food for such individuals (Figure 3b), with 66% (33/50) and 77% (58/75)% of respondents (food allergic individuals and buyers respectively ) citing anaphylactic shock and 55% (99/181) and 65% (98/150) respectively of those citing asthma or breathing difficulties as a symptom being formally diagnosed, compared to 43.5% (134/308) and 54% (130/240) of respondents respectively reporting rash and itching. When the diagnostic status of those reporting symptoms of anaphylactic shock and or asthma or breathing difficulties (symptoms considered to be severe) was compared with those respondents reporting any other symptoms there was a significant association between severity of symptoms and formal diagnosis (P < 0.0001).We recognise that 55% of respondents report no formal diagnosis and this therefore could mean that many do not have IgE-mediated allergy. However whilst this observation is important, it does not detract from the results of the survey, which reflect the characteristics and behaviours of people who consider themselves to have food allergy based upon the general understanding of this term and therefore act on this belief.

### Buying behaviours in relation to food allergy characteristics

**Figure 4:**
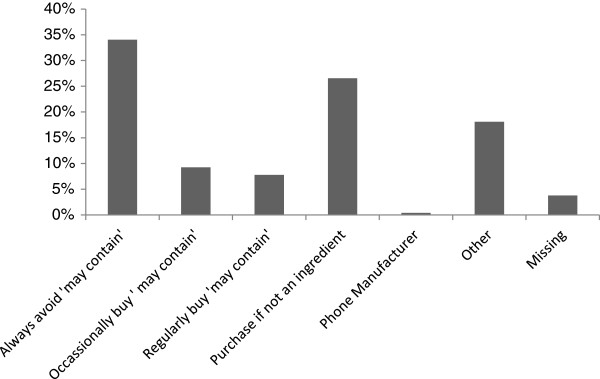
Use of 'May Contain’ labels by allergic consumers and those buying foods for such individuals (n = 949).

**Figure 5:**
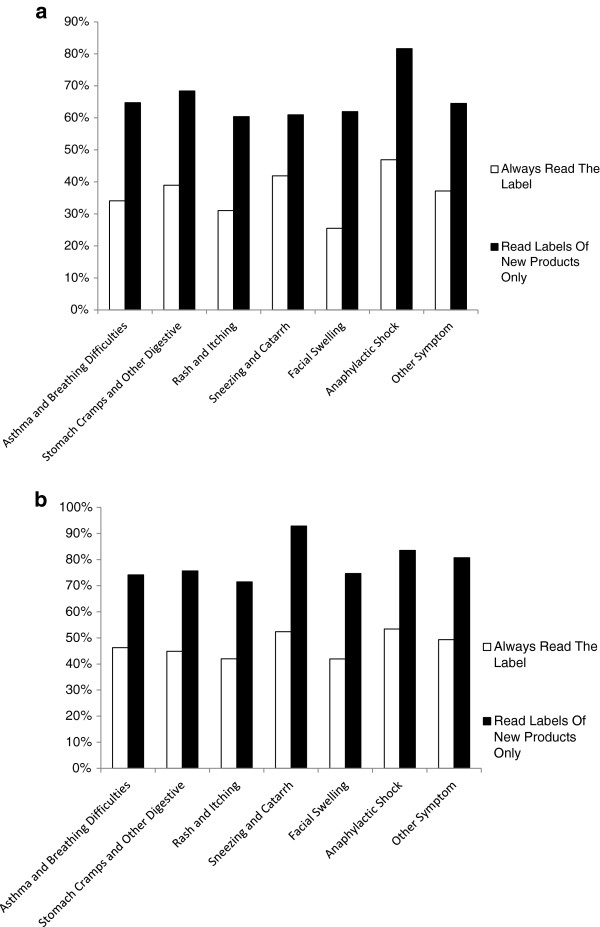
Comparison of buying behaviours reported by respondents reporting different symptoms of food allergy and presented separately for food allergic individuals (a) and those buying foods for such individuals (b).

Only 34% of respondents always read product labels, although this increased to 64% for new products. The same proportion (34%) always avoid products with a relevant 'may contain’ label, while 27% buy such products if the allergen is not listed as an ingredient, and 8% regularly purchase such products (Figure 4). Generally, respondents reporting severe symptoms (asthma, breathing difficulties and anaphylactic shock showed more cautious and vigilant buying behaviour than those citing non-severe symptoms. When the buying behaviours (whether or not labels are always read, only read first time or not always read) of those reporting symptoms of anaphylactic shock and or asthma or breathing difficulties (symptoms considered to be severe) was compared with those respondents reporting any other symptoms, there was a significant association between severity of symptoms and more cautious buying behaviours. Thus respondents reporting severe symptoms were significantly more likely to always read the label (P = 0.0026). A separate analysis of those respondents who cited anaphylactic shock as a symptom revealed that only 47% (23/49) who were food- allergic and 53% (39/73) of those buying foods for such individuals reported that they always read product labels and 82% (40/49) and 84% (61/73) respectively read the labels of new products only (Figure 5a and b).

### Reporting reactions

Respondents were asked the following question to understand how many reactions would come to the attention of public health authorities: “When you or your family member has a reaction to a food, which of the following do you do?” and given the options:

• Always report a reaction no matter how mild.

• Only report a severe reaction, even if managed without visiting a medical practitioner.

• Never report a reaction.

The majority of respondents (52%) never reported reactions, with 34% reporting a reaction when severe and only 6% always reporting a reaction.

Surveys based on self-reporting frequently over-estimate the incidence and prevalence of food allergies, but much depends on how the question is posed. They also have the potential to distort the pattern, compared to the actual one observed in the population of interest. The pattern of self-reported food allergy among respondents in this study closely reflects that described in the scientific literature for Northern Europe in terms of foods implicated and distribution with age, with children being primarily affected by food allergies, egg being the most common food allergen in young children, milk in school-age children and fruit and vegetables in adults (with the latter possibly reflecting cross-sensitisations with pollen allergens in this age group) [25,26]. The pattern of symptoms reported also reflects those in the medical literature with skin being the most common. This suggests that the respondents are a good representation of the food allergic consumer population in GB. Furthermore this population is different from the usually targeted clinic or patient group populations and includes people with allergies to a range of allergens; it could therefore be considered to be more representative of the population at large.

As has been highlighted by the medical community in GB [22-24] access to allergy services needs to be improved and this is reflected in the results of this survey where a minority report food allergy being formally diagnosed. We note that respondents reporting more severe symptoms were more likely to report a food allergy as being formally diagnosed. Nevertheless it is concerning that a large proportion of respondents reporting severe symptoms such as breathing difficulties and anaphylactic shock had not been formally diagnosed and could be reasonably considered to most likely not have received formal counselling regarding allergen avoidance. Taken together, these findings imply that the prevalence of food allergies in GB is most likely under-estimated, as the majority of respondents report no formal diagnosis, and more severe reactors are over-represented in GB clinic populations, as might be expected. This has potential implications for work underway to define 'action levels’ for the risk management of food allergens in food manufacturing, as such levels are based upon data derived from clinic populations. It is positive to note however that individuals with more severe symptoms are most likely to be protected by approaches where action levels are derived from clinic data rather than a random population sample.

A substantial minority of respondents regularly place themselves at risk when purchasing food products, with only approximately a third reading product labels every time and approximately two thirds when they buy a new product. In addition, although those with a formal diagnosis were more cautious, even among respondents reporting anaphylactic shock only approximately 50% always read labels, whilst approximately 80% read the labels of new products. When specifically questioned about 'may contain’ labelling consumers always avoiding products with such labels were also in the minority.

Patient advocacy groups, such as the Anaphylaxis Campaign strongly recommend to their members that they always read the full ingredient list, as well as any precautionary statement. The failure of most food-allergic consumers, including those with potentially severe symptoms, to read labels on every occasion highlights the importance of food manufacturers thinking beyond legal compliance when designing labels to communicate with their allergic consumers. This finding clearly illustrates that where an allergen is formulated into a product that previously did not contain it, simply adding the allergen to the ingredient list may not sufficiently protect the allergic consumer. In such cases the addition of the allergen should be flagged on the front of the pack to alert consumers such a change e.g. using the wording 'New recipe’ or 'Now Contains’ [27].

Noimark et al. (2009) reported that in a survey of parents of nut allergic children attending a tertiary paediatric allergy clinic in the UK, more than 80% would avoid a product labelled 'may contain nuts’ or 'not suitable for nut allergy sufferers’, however only approximately 60% would not purchase a product labelled 'may contain traces of nuts’ [15]. These response rates were similar to a study among a Japanese population with self-reported severe food allergies [28] but are higher than found in this survey. However this is perhaps not unexpected given that in the case of Noimark et al. those questioned were parents of nut allergic children reaching a tertiary referral clinic and in the case of Imamura et al., individuals with severe food allergies. Again it is noteworthy that even among the potentially more aware consumers at higher risk, a substantial minority still purchase products with a 'may contain’ label. Similar findings regarding food allergic-consumers’ attitudes to labelling, and in some cases ignoring of labels leading to accidental exposures, have been reported in other countries, such as Canada [16,17] and the US [18] illustrating that this behaviour is potentially global in nature. Limitations and consequences of the current approach to 'may contain’ labelling for food allergens have been considered by groups such as Turner et al. (2011) and are consistent with the findings of this survey [29]. It is clear that there is a need for agreed, accepted standards for determining when precautionary allergen labelling should be applied and there is a great deal of time and effort currently being invested in addressing this challenge [19-21].

As indicated by the reported lack of formal diagnosis and publicised issues with access to allergy services in the UK, reaching and educating food-allergic individuals in GB regarding any changes in food allergy labelling will be challenging. Indeed as reported by Jones et al. (2010) 'GPs may benefit from education and ongoing decision support and be supported by public education on the nature of allergy’ [24]. Thus alternative education delivery, beyond that via National Health Service (NHS) clinics and dieticians, may prove more effective in the UK.

The large majority of respondents stated that they never report reactions and the likelihood of reporting was associated with symptom severity. Assessing the impact of future changes in risk management measures can probably not be achieved by simple monitoring, but will require more focussed studies.

## Conclusions

This study confirms that the current application of precautionary labelling to mitigate and communicate the risk from allergenic foods is of limited effectiveness and with the reported increasing prevalence of food allergies, and until effective treatments become available, it is important that labelling is as robust and effective as possible, accurately and clearly communicating risk to the allergic population.

## Competing interests

Stella Cochrane, René Crevel and David Sheffield are employees of Unilever.

## Authors’ contributions

SC, HG and RC were involved in the design of the study questionnaire, data interpretation and drafting, revising and final approval of the manuscript. DS was involved in the data analysis and revising and final approval of the manuscript. All authors read and approved the final manuscript.

## Supplementary Material

Additional file 1 Details of all questions as put to questionnaire respondents. Click here for file
