# Supplementary material for: Characteristics and purchasing behaviours of food-allergic consumers and those who buy food for them in Great Britain
Source: Clin Transl Allergy. 2013 Sep 23;3:31. doi: 10.1186/2045-7022-3-31 (PMC3850942; doi:10.1186/2045-7022-3-31)
Supplement: Additional file 1 — Details of all questions as put to questionnaire respondents. [file 2045-7022-3-31-S1.doc]

1. ***“Symptoms of allergic reactions to foods can include mouth itching, swelling of the lips, face, throat, mouth and/or tongue, rashes, asthma, or even collapse and unconsciousness. These reactions almost always happen within two hours of eating the food***

Have you ever suffered any such reaction to any foods, or are you the main food buyer (for example a parent or partner) for someone who has suffered from such a reaction? Please tell us even if the reaction was relatively mild.

[Please tick all that apply]

1. Sufferer
2. Buyer
3. Neither
4. What type of reaction(s) have you/ your family member suffered?

[Please tick all that apply]

1. Breathing Difficulties
2. Asthma
3. Stomach Cramps
4. Other Digestive Problems
5. Rash
6. Itching
7. Sneezing
8. Facial Swelling
9. Swollen Glands
10. Migraine / Headaches
11. Catarrh
12. Aching Joints / Muscles
13. Panic Attacks
14. Hyper Activity
15. Anaphylactic Shock
16. Other
17. Who in your household suffers from food allergies?

[Please tick all that apply]

1. Myself
2. My spouse / partner
3. Other adult(s)
4. Baby, under 1
5. Toddler, 1 to 2
6. Young child, 3 to 4
7. Primary school child, 5 to 10
8. Secondary school child, 11 to 18
9. Which of the following food groups are / have been responsible for causing the allergic reaction?

[Please tick all that apply]

1. Cow’s milk, butter, cheese or other products made with cow’s milk
2. Soya
3. Peanuts
4. Other nuts such as Brazil nuts, hazelnuts, walnut and pecan
5. Cereals such as wheat, rye, barley, oats, maize or rice
6. Eggs
7. Fish and Shellfish
8. Fruit and Vegetables
9. Other (please specify on the next page)
10. What other food group has caused an allergic reaction?

[Please type in]

1. Have you/ your family member ever had a food allergy formally diagnosed?

- Yes
- No

1. Who have you / your family member had a food allergy diagnosed by?

[Please tick all that apply]

1. Health visitor
2. GP
3. NHS specialist in a hospital / clinic
4. Dietician
5. Private allergy specialist
6. Other
7. Have you / your family member ever had a blood test in a hospital / clinic to test for your food allergy?

- Yes
- No

1. What was the result of the blood test?

[If you / your family member have been tested more than once, please think of the most recent occasion]

- - Positive
  - Negative

1. Have you / your family member ever had a skin prick test in a hospital / clinic to test for your food allergy?
   - Yes
   - No
2. What was the result of the skin prick test?

[If you / your family member have been tested more than once, please think of the most recent occasion]

- Positive
- Negative

1. Which, if any, of the following have you / your family member done to seek advice on food allergies?

[Please tick all that apply]

1. Telephoned manufacturers’ careline (telephone number on the food packet)
2. Telephoned supermarket carelines
3. Visited manufacturers’ websites for advice
4. Visited supermarket websites for advice
5. Registered with a manufacturer
6. Registered with a supermarket
7. Visited The Anaphylaxis Campaign website
8. Visited other websites focusing on food allergy
9. Joined the Anaphylaxis Campaign
10. None of these
11. Have you ever used a careline phone number on a packet of food to get information regarding allergens or to report a reaction to a food?

[Please tick all that apply]

1. Have used a careline to get information about allergens
2. Have used a careline to report a reaction
3. Neither of these
4. When you / your family member has a reaction to a food, which of the following do you do?

- Always report a reaction no matter how mild
- Only report a severe reaction, even if managed without visiting a medical practitioner
- Never report a reaction

1. To which, if any, of the following have you / your family member reported a reaction?

[Please tick all that apply]

1. Food manufacturer
2. GP
3. Hospital
4. Dietician / Nutritionist
5. Other
6. Have not reported to anyone
7. Have you / your family member ever participated in a food challenge study in a hospital?
   - Yes
   - No
8. Which of the following types of treatment have you / your family member been provided with for your food allergy?

[Please tick all that apply]

1. Inhaler
2. Anti-histamine
3. Injectable adrenalin, e.g. Epipen / Anapen
4. None of these
5. Do you regularly shop for food?

- Yes
- No

1. Which, if any, of the following precautions do you take when shopping for food?

[Please tick all that apply]

1. Avoid shopping in supermarkets
2. Buy food from special ‘free from’ ranges from supermarkets
3. Buy food from special ‘free from’ ranges via the Internet/Mail order
4. Always read the labels even when buying foods you have bought before
5. Always read the labels when buying products for the first time
6. Avoid buying unwrapped food from the deli counter
7. Avoid buying bread from the bakery department
8. Avoid buying from local bakers and other food shops
9. Other (please specify on next page)
10. None of these
11. What other precautions do you take when food shopping?

[Please type in]

1. When buying foods for yourself / your family member who has a food allergy, which, if any, of the following do you do?

- Always avoid products where the allergen of concern is indicated on a ‘may contain’ type label
- Occasionally buy products where the allergen of concern is indicated on a ‘may contain’ type label
- Regularly take calculated risks and therefore occasionally buy products with these labels
- Purchase the product as long as the allergen is not a listed ingredient
- Phone the manufacture for more information
- Other

1. If you noticed that a food which you / your family member had consumed safely before had a ‘may contain’ type label, but the allergen was not listed as an ingredient, which of the following would you do?

- Continue to buy the product
- Stop buying the product
- Don't know
